# Patient-Specific 3D-Printed Models in Pediatric Congenital Heart Disease

**DOI:** 10.3390/children10020319

**Published:** 2023-02-07

**Authors:** Zhonghua Sun

**Affiliations:** 1 Discipline of Medical Radiation Science, Curtin Medical School, Curtin University, Perth, WA 6845, Australia; z.sun@curtin.edu.au; Tel.: +61-8-92667509; 2 Curtin Health Innovation Research Institute (CHIRI), Curtin University, Perth, WA 6845, Australia

**Keywords:** three-dimensional printing, congenital heart disease, children, model, personalized medicine, application

## Abstract

Three-dimensional (3D) printing technology has become increasingly used in the medical field, with reports demonstrating its superior advantages in both educational and clinical value when compared with standard image visualizations or current diagnostic approaches. Patient-specific or personalized 3D printed models serve as a valuable tool in cardiovascular disease because of the difficulty associated with comprehending cardiovascular anatomy and pathology on 2D flat screens. Additionally, the added value of using 3D-printed models is especially apparent in congenital heart disease (CHD), due to its wide spectrum of anomalies and its complexity. This review provides an overview of 3D-printed models in pediatric CHD, with a focus on educational value for medical students or graduates, clinical applications such as pre-operative planning and simulation of congenital heart surgical procedures, and communication between physicians and patients/parents of patients and between colleagues in the diagnosis and treatment of CHD. Limitations and perspectives on future research directions for the application of 3D printing technology into pediatric cardiology practice are highlighted.

## 1. Introduction

Three-dimensional (3D) printing technology is being increasingly used in the medical field, with studies documenting its application in a range of areas, from its original application in maxillofacial and orthopedic surgery to cardiovascular disease [1,2,3,4,5,6,7,8,9,10,11,12]. Extensive research has proved the usefulness and established the clinical value of 3D-printed models in maxillofacial surgery and orthopedics. Meanwhile, the investigation of 3D-printed models for use in cardiovascular disease is an emerging but rapidly growing area with research that has confirmed its great potential in this domain. High-quality 3D-printed models derived from cardiac images such as computed tomography (CT), magnetic resonance imaging (MRI) and echocardiography are highly accurate in replicating normal cardiac anatomy and pathology, thus serving as a valuable and complementary tool to current image visualizations in the diagnostic assessment of cardiovascular disease [13,14,15,16,17,18,19,20,21,22,23,24,25,26,27,28,29,30,31,32,33,34,35].

Of various cardiovascular abnormalities, congenital heart disease (CHD) represents a very challenging area due to the complexity of the congenital anomalies, the broad spectrum of conditions and the high variability between individuals. In the pediatric sub-specialization, a basic understanding of common CHD is necessary and plays an important role in clinical decision making and patient management. However, the standard visualization techniques, including cardiac CT, MRI and echocardiography, are limited in their ability to convert 2D images into a 3D object on a flat screen and so do not allow full comprehension of complex intracardiac anatomy and defects [19,36]. To overcome these limitations, 3D-printed physical models demonstrate superior advantages and strengths over the current image visualizations by providing realistic 3D representations of the spatial relationships between these cardiac structures and abnormal changes that are difficult to acquire on traditional 2D and 3D image reconstructions. In addition to its enhancement in understanding complex anatomy, 3D-printed heart models serve as a valuable tool to guide surgical planning, to train junior or inexperienced pediatric residents, and to educate healthcare professionals or parents of patients [13,14,15,16,17,18,19,20,21,22,23,24,25,26,28,29,30,31,32,33,34,35]. This review aims to provide a summary of the current applications of 3D-printed models in pediatric CHD, and highlights limitations and future research directions about 3D printing in pediatric cardiology practice. The reason we focus on pediatric CHD is due to the increased use of 3D printing technology in CHD, in particular in the field of pediatric CHD where the rarity of pathology and the smaller size of the patients present challenges for clinicians in the diagnosis and management of CHD conditions. Three-dimensional-printed heart models with the unique advantage of an improved demonstration of complex cardiac defects at high fidelity could overcome these challenges. It is expected that this comprehensive review of the current literature on 3D printing in pediatric CHD will provide readers with a useful resource about the clinical value and applications of 3D-printed models in pediatric CHD.

## 2. Generation of 3D-Printed Heart Models

**Figure 1:**
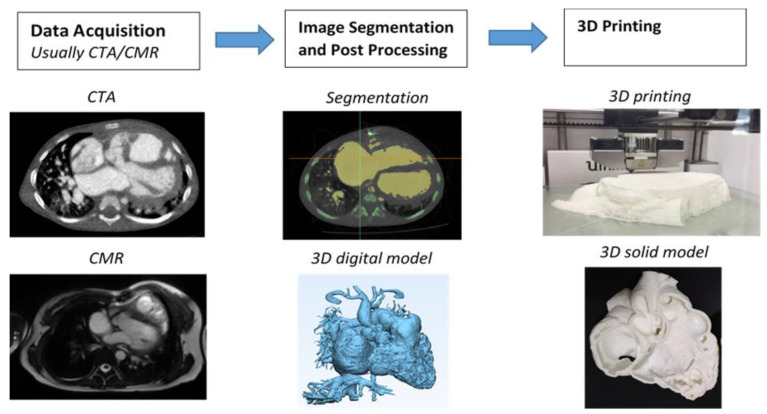
Steps to generate 3D printed heart models. CTA-computed tomography angiography, CMR-cardiac magnetic resonance. Reprinted with permission under the open access from Sun et al. [7].

The steps from processing the original 2D digital imaging and communications in medicine (DICOM) images to 3D volume data, and from there to the segmentation of the image is well explained in the literature, and usually involves semi-automatic along with manual editing processes. Figure 1 shows a flow chart representing the creation of a 3D-printed heart model from an example of cardiac CT images. In general, to ensure the quality of the 3D-printed model, high resolution imaging data comprises an essential component in the production of an accurate 3D-printed model. Cardiac CT is the most commonly used modality in 3D printing due to its high spatial resolution, although cardiac MRI and echocardiography are also used in some studies when printing CHD models [37].

**Figure 2:**
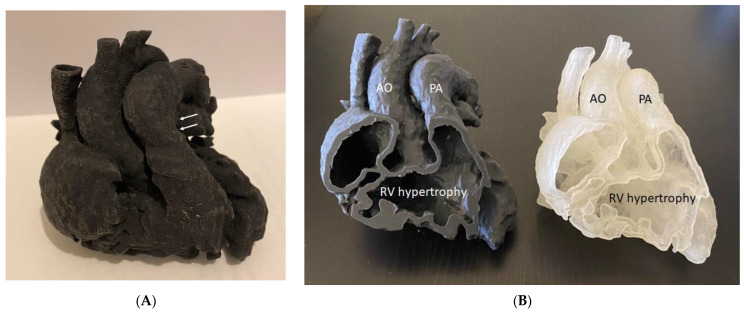
Three-dimensional-printed heart model of a case with Tetralogy of Fallot (ToF). (**A**): The model was printed in one piece. (**B**): The model was printed in two halves (with the same material, Agilus30, but different colors) showing the internal cardiac chambers and vascular abnormalities. Arrows indicate the pulmonary artery stenosis. AO—aorta (overriding aorta); PA—pulmonary artery; RV—right ventricle.

**Figure 3:**
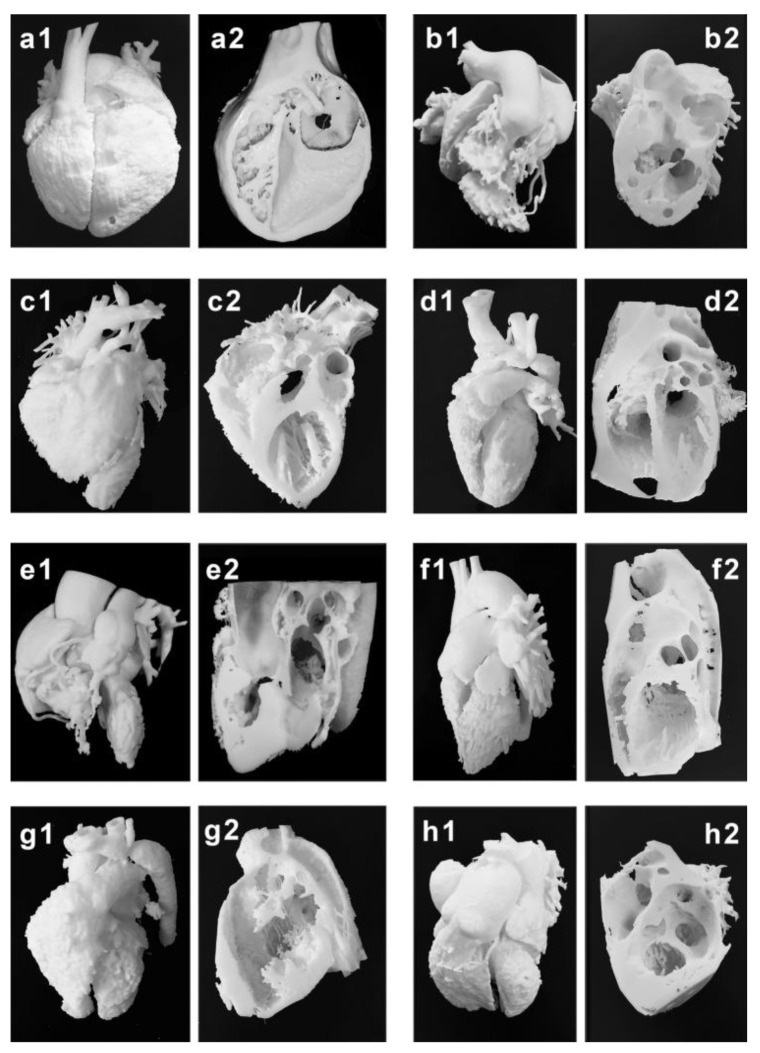
Three-dimensional printing of the blood pool and myocardium (showing inside views of cardiac structures at different angles) models for eight typical CHD cases (**a**–**h**). (**a1**–**h1**) are blood pool models, while (**a2**–**h2**) are myocardium models showing internal cardiac structures. Case 1: congenital corrected transposition of the great arteries. Case 2: double outlet right ventricle. Case 3: Williams syndrome. Case 4: coronary artery fistula. Case 5: Tetralogy of Fallot. Case 6: patent ductus arteriosus. Case 7: coarctation of the aorta. Case 8: ventricular septal defect. Reprinted with permission under the open access from Liang et al. [38].

Mimics is the most commonly used commercial software tool in image post-processing of 3D datasets for 3D printing, while 3D Slicer is an open-source tool commonly used for segmentation of volumetric data for 3D printing. Usually, hollow heart models are generated to show both outside and inside views of cardiac anatomy and pathology as shown in Figure 2, while blood pool models can also be printed to accurately demonstrate the spatial relationships between cardiac structures [38] as shown in Figure 3.

## 3. Accuracy of 3D-Printed Heart Models Derived from Imaging Modalities

Three-dimensional-printed models must accurately replicate anatomical details with high accuracy so that the models can be reliably used for different purposes. Current research has shown a very small difference between 3D-printed models and original source imaging data, whether they are acquired with CT, echocardiography, MRI or rotational angiography. Table 1 summarizes the findings of dimensional measurements between a 3D-printed model and the original source images based on the current literature [39,40,41,42,43,44,45]. It is noted that the mean difference in dimensional measurements between 3D-printed models and original images is less than 0.5 mm indicating the high accuracy of the 3D models, with excellent correlations between different observers and measurement approaches.

## 4. Three-Dimensional-Printing Materials for Printing Patient-Specific Models

**Figure 4:**
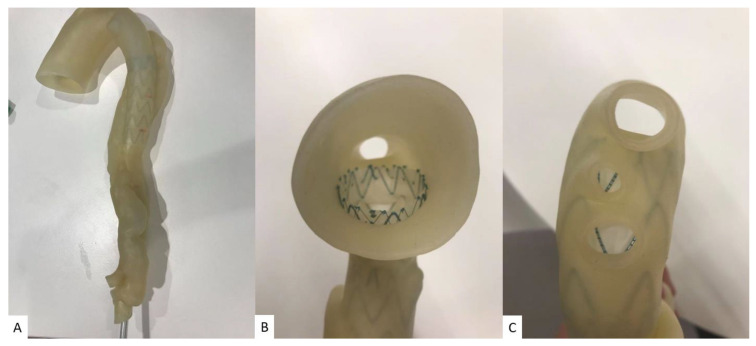
Three-dimensional-printed type B aortic dissection model for simulation of endovascular aortic repair of type B aortic dissection. The printing material was Visijet CE-NT A30. An aortic stent graft was placed into the true lumen of the 3D-printed model (**A**) with axial (**B**) and caudal views (**C**) showing the proximal aortic arch and vessels. Reprinted with permission under the open access from Wu et al. [46].

While commonly used 3D printers including fused deposition modelling (FDM), stereolithography (SLA), polyjet and selective laser sintering (SLS) are the most well known printers in the literature. Printing materials play an important role in determining the final printed models that are most appropriate to serve the purpose of different applications. The models can be printed with rigid materials (such as polylactic acid, rigid resin and rigid photopolymer (VeroClear)) as shown in many early studies [37], however, for printing heart or vascular models, soft and elastic materials with tissue properties similar to normal heart or vascular tissues are more important for the production of realistic heart models. Elastic materials include thermoplastic polyurethane (TPU), Tango materials such as TangoPlus, TangoGray, and Agilus A30 and Visiject CE-NT, and show great promise in printing CHD models. This is especially important when using 3D-printed models to simulate cardiac surgery or interventional cardiac procedures, as operators need to acquire similar tactile experience when performing simulation procedures on realistic 3D printed models [37]. Figure 4 is an example of a 3D-printed aorta model using Visijet CE-NT A30 material, a material which is soft and elastic and has the same tissue properties as cardiovascular tissues [46].

**Figure 5:**
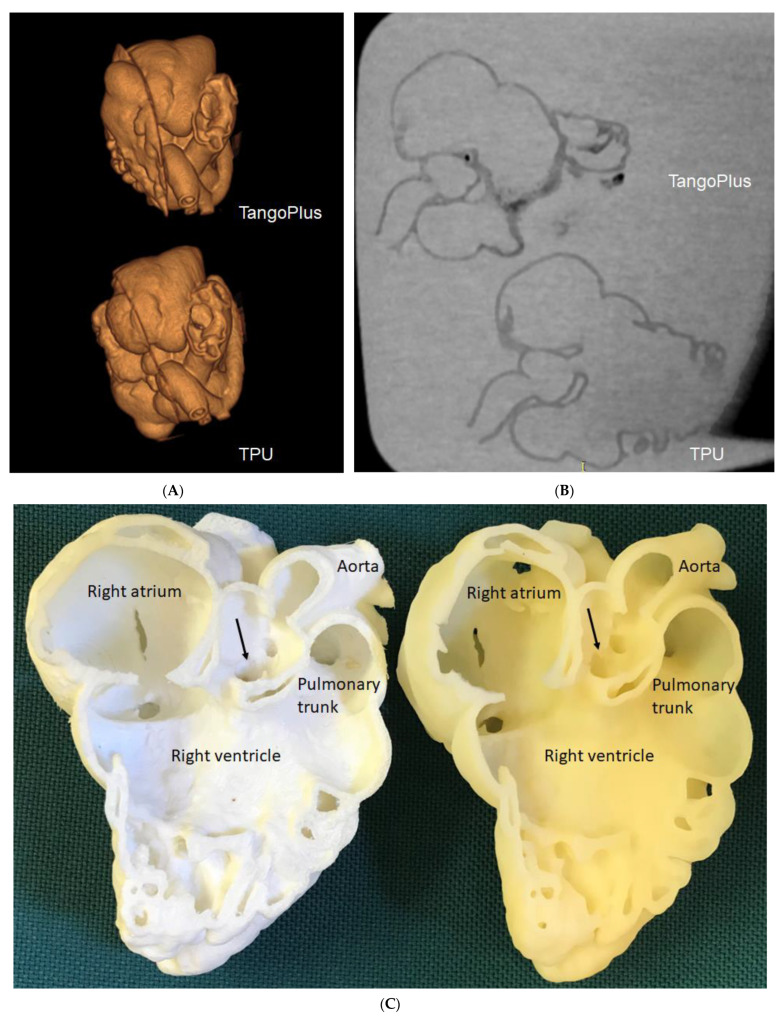
Three-dimensional-printed CHD models with the use of different materials for comparison of model accuracy. (**A**): Three-dimensional CT volume rendering of the 3D-printed models showing similar anatomical details. (**B**): Two-dimensional axial CT views of the 3D printed models. (**C**): Inside view of cardiac chambers and aortic structures on both models. The white model is printed with TPU, while the yellow model is printed with TangoPlus. Arrows refer to the subaortic ventricular septal defect.

The cost of the printing materials is a key factor that could hinder the widespread use of 3D printing in clinical practice. It has been reported that the use of low-cost printed models is feasible and accurate for the determination of CHD cases [19,47,48]. A recent study has further confirmed the accuracy and clinical value of using low-cost 3D printed CHD models [49]. Lau et al. selected a CHD case of double outlet right ventricle (DORV) with models printed using low-cost TPU 95A (AUD $50) and high-cost TangoPlus (AUD $300), respectively. Both materials are flexible, although the TPU material is not as flexible as TangoPlus. Contrast-enhanced CT scans were performed on these 3D-printed models and measurements were conducted at 10 different anatomical locations to compare the model’s accuracy to that of the original CT images (Figure 5). Their results show a strong correlation between measurements from both 3D-printed models and original CT images. Further, these two models were ranked with the same scores by clinicians, wherein both the authors of the study and then the clinicians perceiving the same clinical value or efficacy. Gomez-Ciritza et al. reported the same findings based on their seven-year experience of using low-cost 3D-printed heart models [21]. The authors produced 138 models based on cardiac CT or MRI images in patients with CHD with the mean cost of each model being 85 euro. These 3D-printed heart models were used in different applications from surgical planning of CHD and interventional procedures to education and communication with patients, colleagues and relatives.

The specific purpose of the 3D-printed CHD models determines the selection of appropriate printing materials. For example, if 3D-printed models are used for the educational purpose of learning cardiac anatomy and pathology, rigid or relatively soft and low-cost materials are acceptable. If the 3D-printed models are primarily used for pre-surgical planning or simulation of complex cardiac procedures, soft and elastic materials (or high-cost materials) with tissue properties similar to normal cardiovascular tissues are necessary to allow users to gain learning experience on the 3D models prior to operating on patients.

## 5. Educational and Clinical Value of 3D-Printed Heart Models in CHD

Three-dimensional-printed models are useful in medical and clinical education as the 3D-printed personalized models demonstrate superior advantages over current image visualization tools for the enhancement of students and medical graduates’ understanding of the complex 3D cardiac anatomy and pathology. Of the current applications of 3D printing in cardiovascular disease, 3D-printed CHD models represent the most common application in medical education according to the current literature [37,50,51,52,53]. Studies have provided strong evidence in support of the use of 3D-printed models in CHD education, both for medical students, medical graduates (pediatric residents) and healthcare professionals.

**Figure 6:**
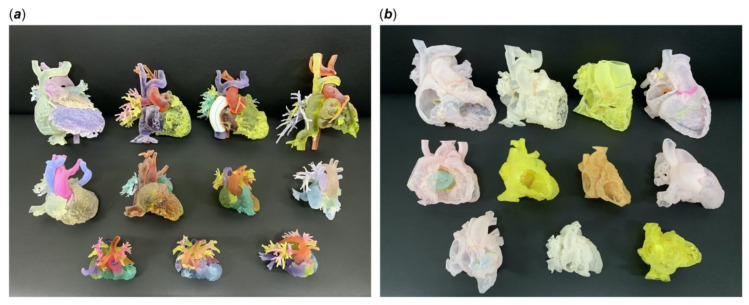
Three-dimensional-printed models of different kinds of complex CHD. (**a**) Blood pool models, (**b**) hollow models. Reprinted with permission from Lee C and Lee J [55].

**Figure 7:**
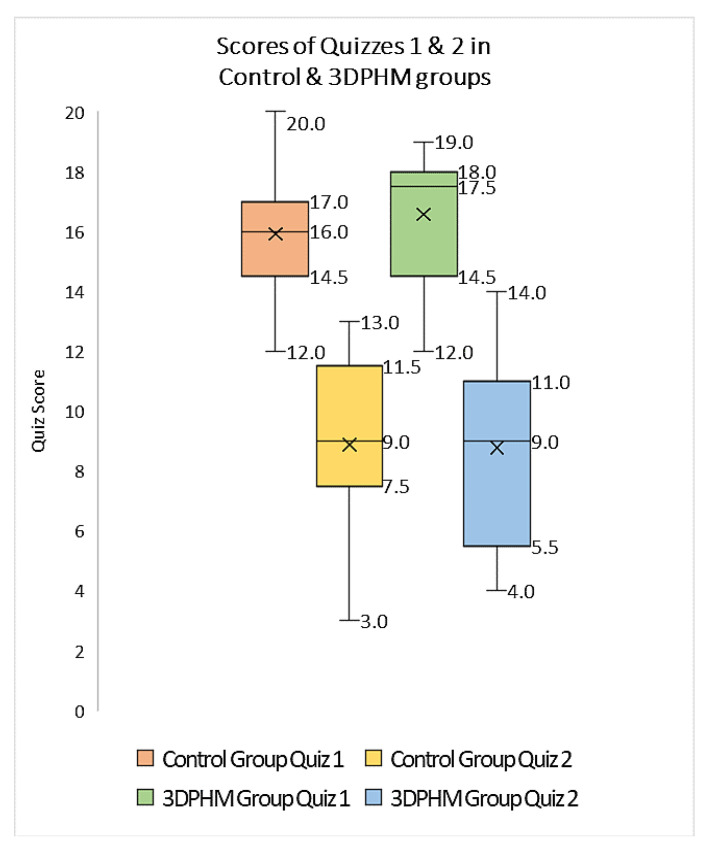
Boxplot showing the scores (out of 20) of quizzes one and two in 3D printing and control groups for 3DPHM-3D printed heart model. Reprinted with permission under the open access from Lau and Sun [25].

Table 2 summarizes representative studies reporting the educational and clinical value of 3D-printed models in pediatric CHD. These studies have shown that 3D-printed heart models significantly enhance medical students, pediatric residents, clinicians and pediatric cardiac nurses’ knowledge in learning normal cardiac anatomy and CHD (Figure 6) [25,26,28,29,30,31,33,34,35,38,54,55]. Three-dimensional-printed CHD models were rated highly in terms of their accuracy and satisfaction scores by the study or experimental groups when compared with the control groups, and this is particularly obvious when assessing the complex CHD in comparison with simple CHD [34]. In their recent report, Lau and Sun did not show significant improvements in knowledge retention among second and third year medical students when 3D-printed CHD models were compared with the current teaching methods using DICOM images and digital 3D heart models, although slightly higher scores were achieved in the 3D printing group (Figure 7) [25].

**Figure 8:**
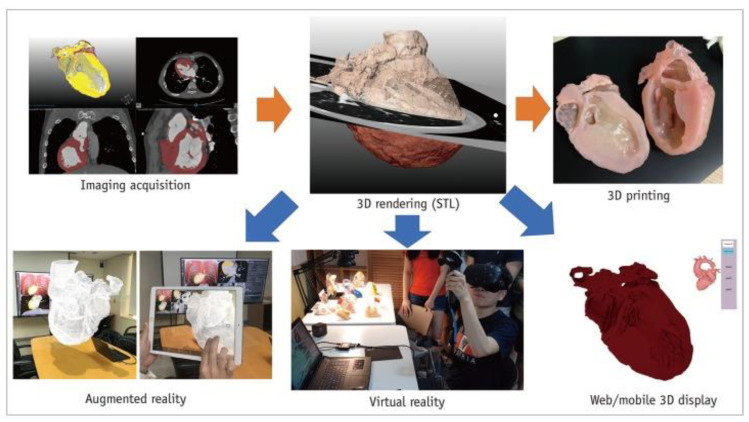
Workflow of advanced visualization technology. Segmented 3D volume data can be used for 3D printing, virtual reality, augmented reality and interactive or mobile 3D displays. Reprinted with permission under the open access from Goo et al. [60].

Another innovative tool in CHD education is the use of either virtual reality (VR), augmented reality (AR) or mixed reality (MR) as an alternative to 3D-printed models in education and pre-surgical planning of CHD. A user is immersed in a virtual environment during VR visualization, while AR is different from VR with virtual objects overlaying on the real world. MR extends the ability of VR and AR by allowing the user to interact with combined virtual and real objects (such as 3D-printed physical models) [56,57,58,59,60]. Figure 8 shows the workflow of these visualization technologies including 3D printing, VR and AR.

VR, AR and MR can be used for education, planning and simulation of CHD surgical procedures. In their recent review, Barteit et al. analyzed 27 studies with applications of AR and MR mainly in surgery planning (48%) and anatomy learning (15%) [61]. Lau et al. also reported the similar value of VR as opposed to 3D printing for CHD medical education through the assessment of four selected CHD cases by 29 participants [30]. VR was ranked as the most useful tool in these two areas when compared with 3D-printed models, though no significant differences were found between these two modalities. More than 70% of participants indicated that VR and 3D-printed models offered additional benefits over conventional image visualizations. In their randomized controlled trial (RCT), Patel et al., compared VR with desktop 2D views of CHD cases, with 24 and 27 participants allocated to each group, respectively [58]. Participants’ impressions of CHD education with the aid of VR was 29% higher than the desktop group (*p* = 0.01). This study shows that VR may increase learner’s engagement in understanding CHD.

**Figure 9:**
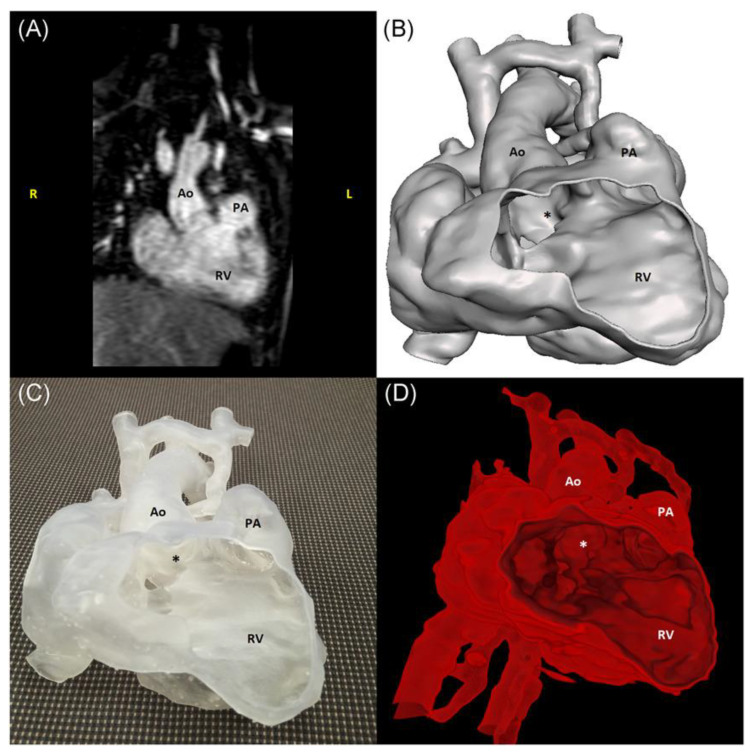
Images of a case with DORV with hyperplasia of the left ventricle and subaortic VSD: CMR scan (**A**), 3D PDF reconstruction (**B**), 3D-printed model (**C**), a screenshot of the DIVA software (**D**). * refers to ventricular septal defect. Ao—aorta; CMR—cardiac magnetic resonance; DORV—double outlet right ventricle; PA—pulmonary artery; RV—right ventricle; VSD—ventricular septal defect. DIVA refers to the user-friendly software for VR visualization. Reprinted with permission from Raimondil et al. [62].

Factors that could limit the use of 3D printing technology on a daily basis include image post-processing and segmentation time, as well as printing cost issues, hence, VR could be a potential alternative to 3D printing in medical education for CHD, and this has been confirmed by Raimondil and colleagues [62]. Investigators compared 3D printing, 3D PDF and VR in three cases of CHD and asked a senior pediatric cardiac surgeon to assess the performance of these three modalities in visualizing anatomical structures. The median post-processing time to generate VR models was only 5 min which is significantly shorter than the 8 h for 3D models (3D printing and 3D PDF) (Figure 9). Their study shows the feasibility of generating VR views directly from the raw imaging data without undergoing any preliminary segmentation process. This could be a promising technique for routine clinical application where 3D printing facilities are not available.

## 6. 3D-Printed Models in CHD: Clinical Applications

Clinical value of 3D-printed models in CHD has been confirmed by a number of studies which are most commonly based on single center experiences or some case reports [21,23,39,63,64,65,66,67,68,69,70,71,72,73,74,75,76,77,78,79,80,81,82,83]. The clinical applications comprise three main areas, including pre-surgical planning of congenital heart surgery, simulation or training of congenital heart surgery procedures, and enhancing physician–patient or between colleagues’ communication through use of personalized 3D-printed heart models. 

### 6.1. Pre-Surgical Planning of CHD Surgery

**Figure 10:**
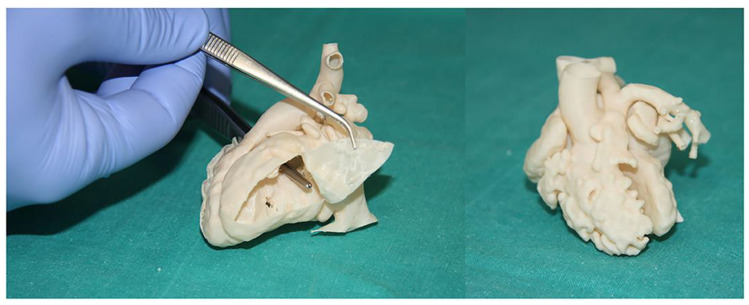
Surgical and interventional planning on 3D-printed heart models. DORV case, internal vision from the left ventricle (left). DORV (another case), external view (right). DORV-double outlet right ventricle. Reprinted with permission under the open access from Gomez-Ciriza et al. [21].

Table 3 summarizes the current literature regarding the use of 3D-printed models in the surgical treatment of pediatric patients with CHD. These studies showed that the use of 3D-printed heart CHD models assisted pre-surgical planning, with up to 50% of surgical decisions changed by 3D-printed models according to a multi-center study [40]. Similarly, a number of studies reporting their single center experience with inclusion of either small or large number of cases also confirmed the usefulness of 3D-printed heart models in guiding surgical procedures in the management of pediatric CHD [21,23,63,64,65,66,67]. Two studies by Gomez-Ciriza and Ryan et al. presented their seven- and three-year experiences with more than 100 3D heart models printed in their clinical practices [21,65]. Gomez-Ciriza and colleagues reported similar findings to Valverde et al. [40], in which about 48% of surgical planning was modified with aid of 3D-printed heart models (Figure 10) [21]. In contrast, based on a three-year experience, Ryan et al. did not show significant differences between 3D-printed models (79 models of different CHD types) and standard of care in surgical planning of CHD [65].

### 6.2. Hands-on Surgical Training for Congenital Heart Surgery Procedures and Medical Education

Congenital heart surgery is a technically challenging field of in pediatrics because of the wide variation of CHD, the rarity of each pathology and the smaller size of the patients. These technical challenges place strong demands for the development of appropriate training programs for surgical trainees to acquire technical skills comparable to experienced surgeons. Three-dimensional-printed heart models with high fidelity for replicating complex anatomy serve as a valuable simulation-based training program for surgical trainees to develop their technical skills prior to performing operations on patients. The hands-on surgical training (HOST) course enables participants to perform surgical procedures on 3D-printed models [68,69,70,84]. In their recent review, Hussein et al. analyzed five studies, with three of these using 3D-printed models for the simulation of congenital heart surgery [68,84,85,86]. These studies supported the idea of using 3D-printed models to prepare surgeons for the simulation of various congenital heart surgery procedures with efficacy.

**Figure 11:**
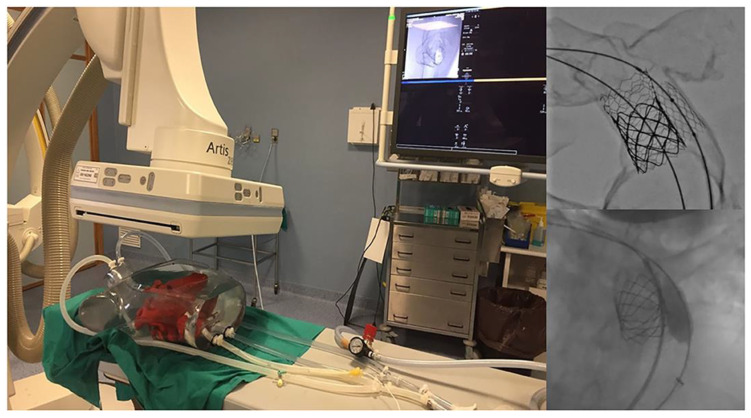
Catheterization applications. Left image shows the fluid inlets and outlets to superior and inferior vena cava and aortic arch/branches, respectively. Interventional planning with 3D-printed model (up right) and real intervention in the patient (down right). Reprinted with permission under the open access from Gomez-Ciriza et al. [21].

**Figure 12:**
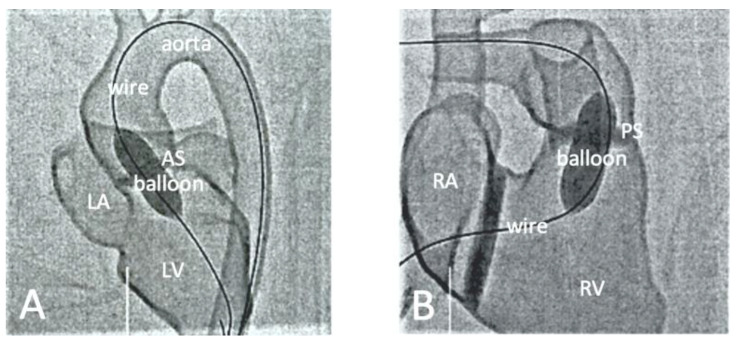
Fluoroscopic visualization of balloon dilatation of valvular stenosis with use of a 3D-printed heart model. (**A**) Balloon dilatation for treatment of a valvular aortic stenosis (AS). (**B**) Balloon dilatation for treatment of a valvular pulmonary stenosis (PS). Reprinted with permission under the open access from Brunner et al. [88].

**Figure 13:**
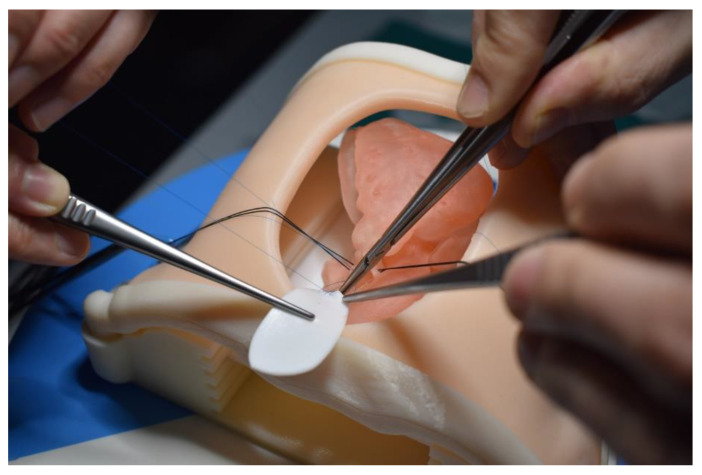
Example of simulation of Tetralogy of Fallot repair on a 3D-printed heart model. Reprinted with permission under the open access from Hon et al. [89].

Table 4 is a summary of studies documenting single center experience of how 3D-printed heart models have been used as a HOST course for congenital heart surgery and medical education [68,85,86,87,88,89,90]. In addition to successfully simulating congenital heart surgery procedures and achieving high satisfaction scores from the participants who acquired the technical skills, 3D-printed models are also useful for simulating interventional cardiac procedures (Figure 11 and Figure 12) [21,88]. The education of medical students and clinicians is another area that has proved the potential value of incorporating 3D-printed models into medical curricula and clinical practice, through HOST courses [84,88,89]. Hon et al. have reported the value of HOST courses for preparing preclinical medical students to work as surgical assistants, as well as consultant cardiac surgeons when performing congenital heart surgery simulations on 3D-printed models [89]. Their results show that early exposure and incorporation of HOST simulation into medical curricula could stimulate the interest of medical students in pursuing highly specialized fields such as congenital heart surgery (Figure 13). Similar findings have been reported by Olivieri et al., who recruited 70 participants for training sessions involving the performance of congenital heart simulation procedures [90]. Three-dimensional-printed models were assessed to be more useful than standard hands-off (8.4 out of 10) training instruments, and served as reliable simulation training tools for congenital cardiac intensive care clinics and enhanced interdisciplinary team communication.

### 6.3. Improving Physician–Patient Communication/Facilitating Communication with Colleagues

Physician–patient communication plays an important role in the clinical setting with patients’ compliance and satisfaction with physicians having an impact on clinical outcomes [91]. Visual aids are commonly used in clinical practice for physicians to explain information to patients regarding the disease condition and treatment options [92]. However, conventional approaches of using 2D diagrams make it difficult for patients to imagine a 3D structure, and this is particularly challenging in CHD as patient and family tend to not possess adequate knowledge and understanding of complex heart anatomy or congenital defects. Three-dimensional-printed models have overcome this limitation by presenting a physically touchable model to the patient with improved understanding of both anatomy and pathology, thus enhancing communication between a physician and their patient and between a physician and their colleagues.

**Figure 14:**
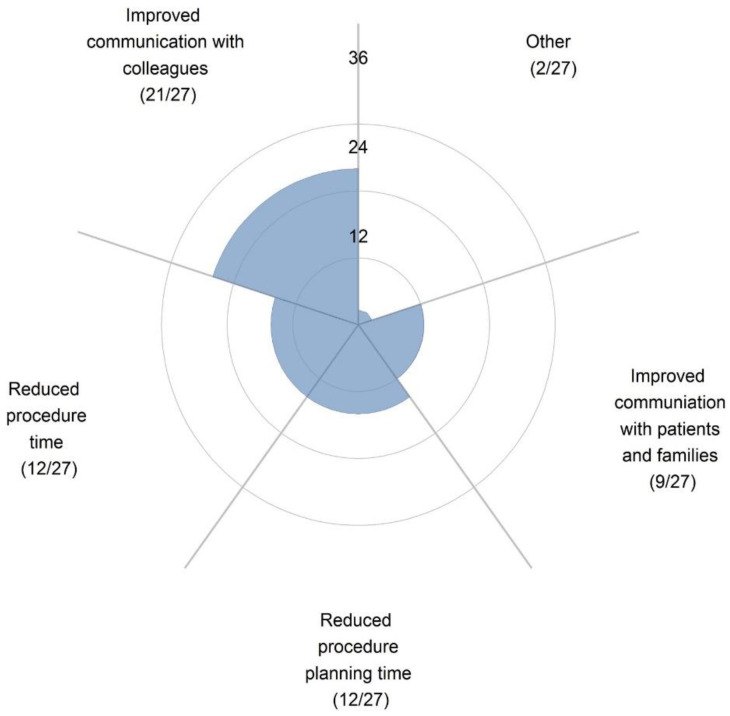
Survey responses show that 3D-printed models were most useful in improving communication with colleagues. Reprinted with permission under the open access from Illmann et al. [50].

A number of studies have reported the value of using 3D-printed models in improving physician–patient communication. In their recent review, Traynor et al. analyzed 19 studies about the use of 3D printing technology in patient communication [93]. Of these studies, seven documented findings on cardiology and cardiovascular surgery [43,50,64,94,95,96,97], of which four studies were conducted by the same research group [94,95,96,97]. Results of these studies confirm that 3D-printed models helped communication with patients/parents and with colleagues (Figure 14), with a significant improvement in knowledge or understanding of the CHD condition, and overall satisfaction. This was confirmed by a recent study comparing 3D-printed heart models with MR in CHD [98]. The 3D-printed heart models were ranked as the best modality by 90% of participants (30 out of 34 physicians surveyed) and the most preferred communication tool with patients when compared with original DICOM and MR (*p* < 0.01).

Deng and colleagues conducted a RCT to study the clinical value of 3D-printed heart models in surgical consent for CHD repair [99]. Guardians of 40 patients with elective perimembranous ventricular septal defect (VSD) repair were invited to participate in the study with 20 allocated to the control and study groups, respectively. The control group received information about VSD condition and surgical indications as well as potential complications with the aid of the current approach using 2D charts, while the study group received the same information but using a 3D-printed model of the heart with VSD to assist explanation of these details. Results show significant improvements in their understanding of the VSD anatomy and the surgical procedures and potential complications (*p* = 0.02 for all of them) in the study group when compared with the control group, although there was no significant difference in overall ratings of the consent process (*p* = 0.09). This study represents the first RCT that quantifies the benefits of 3D-printed models in surgical consent, although further research with inclusion of other types of CHD conditions is needed.

## 7. Limitations, Challenges and Future Directions

In recent years, we have observed significant progress in the use of 3D printing technology in cardiovascular disease, with studies reporting its educational and clinical value in CHD (either educational approaches, or diagnostic methods based on 2D/3D image visualizations and standard training programs). The current evidence has proved that 3D-printed models are highly reliable and accurate in replicating cardiac anatomy and pathology (Table 1). Three-dimensional-printed models can be used as a beneficial tool in treating both adults and children with CHD [50,100]. However, the widespread application of 3D printing technology in pediatric cardiology practice is still hindered to a large extent by some limitations and barriers which need to be considered.

First, access to 3D printing facilities is limited, according to a recent international survey [50]. Illmann et al. surveyed 71 pediatric cardiologists from five continents seeking their opinions on the use of 3D-printed heart models in treating CHD patients and their access to 3D printing technology in their clinical practices. They noticed significant differences in access to 3D printing technology depending on the geographic location of the respondent, with pediatric cardiologists from the USA having more than five-times more access to 3D printing technology than their Canadian colleagues (*p* = 0.004). Financial barriers and preference for standard imaging modalities contribute as main reasons for limited access to 3D printing technology.

The cost of printing materials is another factor that limits its application in many practices. While low-cost 3D-printed models are acceptable for most of the educational and clinical applications [21,49], high-cost 3D-printed models are necessary for the HOST course because of the necessity to print heart models with very soft and flexible materials which enable the participants to develop tactile experience while simulating congenital heart surgery procedures. Agilus30 from Stratasys is currently the preferred material for 3D-printed heart models suitable for CHD surgery simulations (Table 4), but high cost is the main obstacle. Hussein et al. pointed out that 3D-printed model costs per trainee per year up can be as much as US$7500 when adopting the HOST program into congenital heart surgery curriculum [85]. Therefore, further cost reductions are necessary before the use of 3D-printed heart models as a training/simulation tool becomes a reality on a large scale in the near future.

Second, despite significant improvements in 3D printing technology over the last decades, the turnaround time could take several hours or even longer, including steps from initial image post-processing to printing and cleaning of the physical models. This could delay the patient treatment, especially when 3D-printed models are used for pre-surgical planning purposes. When 3D printing facilities are not accessible due to financial barriers, selection of VR or MR could be an alternative to 3D printing technology as these 3D innovative tools provide the same, or improved, advantages in terms of 3D visualization for the more efficient comprehension of CHD as opposed to 3D-printed models [30,61,98]. In their recent study, Lau et al. compared 3D-printed heart models with MR and original DICOM images in two selected pediatric CHD scenarios (ASD and VSD) with the clinical value of these modalities assessed by 34 cardiac specialists and physicians [98]. Their results show that MR was scored as the best modality in most of the clinical applications. This study further proves the potential value of using MR in pediatric cardiology practice when dealing with patients with CHD. Currently, it costs around AUD $600 and AUD $5500 to purchase an Oculus Quest 2 and Microsoft HoloLens for VR and MR demonstrations, respectively. Given the costs associated with 3D printers (including equipment setup, lab space to host the printers and technical support, etc.), printing materials and post-processing steps involved, VR and MR could be a cost-effective alternative to 3D printing technology in medical applications. Future studies are needed to compare the costs of VR/MR (multiple consoles are required for different users) and 3D-printed models (3D models can be handled by multiple students/users at the same time) in these applications. 

Third, the majority of the current studies is based on case series or relatively small sample sizes (Table 2, Table 3 and Table 4), while robust studies such as RCT or multi-center studies are lacking. This could be due to several reasons, including the fact that 3D printing in pediatric cardiology is an emerging area with more evidence needed to prove its educational and clinical value in pediatric CHD before it is widely accepted. As highlighted previously, limited access to 3D printing facilities or financial barriers could explain the fact that most of the studies are case reports or case series. Further, follow-up studies are scarce, hence future studies need to focus on investigations of patient outcomes (mid- to long-term), on the way that 3D-printed models contribute to patient management and on whether it is a cost-effective approach when compared with the current or standard methods in pediatric cardiology practice.

**Figure 15:**
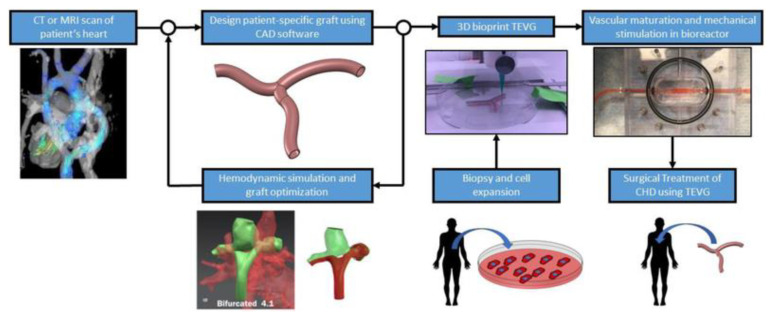
Proposed workflow of patient-specific TEVG fabrication. CAD-computer aided design, TEVG—tissue engineered vascular grafts. Reprinted with permission from Lee et al. [107].

Recent developments in 3D printing technology have made it possible to print biocompatible materials, cells and cardiovascular constructs into 3D tissues so that it now plays an important role in the treatment of pediatric congenital heart defects [101,102,103,104]. Three-dimensional bioprinting offers the potential to develop 3D cardiovascular tissue/organ structures with optimized microenvironments that include cellular morphologies and structures, with research evidence showing the capability of printing complex cardiovascular tissue constructs such as vascular grafts, myocardium and heart valves (Figure 15) [105,106,107]. With the use of 3D bioprinting technology, it is feasible to print a full-size model of the human heart [108,109]. Although these 3D-printed heart chambers had chamber-like contractile function and physiological features, they did not contain all of the main cardiac structures, such as endothelial cells and fibroblasts. Thus, it is still not clinically feasible to directly translate 3D-printed cardiovascular tissues to patient therapy. Further technological advancements in 3D bioprinting could overcome these limitations and challenges and there is no doubt that 3D bioprinting represents an exciting future in treating congenital heart disease and other anomalies.

In summary, 3D printing technology as an exciting and rapidly evolving field has revolutionized our current practice in the diagnosis and management of pediatric patients with CHD. Use of 3D-printed models has augmented the traditional visualization tools when diagnosing and assessing CHD conditions. Research from the current literature has shown that patient-specific 3D printed models serve as a valuable educational tool in learning anatomy and congenital heart defects. Three-dimensional-printed models are of great value in assisting surgical planning and simulation of congenital heart surgery procedures, thus greatly improving surgical care and patient management. Through the use of a hands-on surgical training program, 3D-printed personalized models significantly enhance surgical trainees’ skills and confidence in performing complex cardiac operations. This has a significant clinical impact since successful simulation of congenital heart surgical procedures will result in high operation success rates with lower risks or complications when performing on patients, and thus improving patient outcomes. Three-dimensional-printed models can be used as a more effective tool than the images or diagrams that are currently used during physicians’ communication with patients or with colleagues when dealing with pediatric CHD. To achieve the goal of implementing 3D printing technology into pediatric cardiology practice, a close collaboration between stakeholders and researchers is essential. This ensures that their knowledge and skills in the development of 3D-printed models are fully utilized and maximized to assist the delivery of personalized medicine, thus contributing to an optimized diagnostic strategy and clinical decision-making in pediatric patients with CHD.

## Figures and Tables

**Table 1:** Accuracy of 3D-printed heart model in comparison with original source images according to the current literature. Modified from Lee et al. [39].

Studies	No. of 3D-Printed Models	Comparisons	Mean Difference (mm)	Analysis Method
Lee et al. [39]	3	3D model vs. original CT 3D model vs. CT of 3D model 3D model vs. STL files Original CT images vs. STL files	0.21 ± 0.37 mm −0.11 ± 0.47 mm 0.1 ± 0.28/ 0.17 ± 0.48 mm 0.12 ± 0.23/ 0.12 ± 0.25 mm	Pearson’s correlation/ Bland–Altman plot
Valverde et al. [40]	40 (20 selected for accuracy comparison)	3D model vs. both CT and MRI 3D model vs. original CT 3D model vs. original MRI	0.27 ± 0.73 mm −0.16 ± 0.85 mm −0.30 ± 0.67 mm	Bland–Altman plot
Olejník et al. [41]	8	CT images vs. STL	0.19 ± 0.38 mm	Bland–Altman plot
		3D model vs. in vivo	0.13 ± 0.26 mm	
Olivieri et al. [42]	9	3D model vs. echo	0.4 ± 0.9 mm	Pearson’s correlation/ Bland–Altman plot
Lau et al. [43]	1	3D model vs. CT	0.23 mm	Pearson’s correlation
Mowers et al. [44]	5	2D echo vs. digital 3D	0 mm	Pearson’s correlation/ Bland–Altman plot
		2D echo vs. 3D model	0.3 mm	
Parimi et al. [45]	5	3D model vs. rotational angiography	No significant difference between 3D models and biplane angiography measurements (*p* = 0.14)	Pearson’s correlation/ Bland–Altman plot

DICOM—digital imaging and communications in medicine, CT—computed tomography, MRI—magnetic resonance imaging, STL—standard tessellation language.

**Table 2:** Application of 3D-printed models in medical and clinical education. Modified from Sun and Wee [37].

Author	Study Design	Sample Size and Participants	Original Data Source	Application in CHD	Key Findings
Lau & Sun [25]	Cohort study	53 medical students	CT	ASD, VSD, ToF and DORV.	Slightly higher scores were achieved in the 3D-printed model group than those in the control group (7.79 ± 2.63 vs. 7.04 ± 2.64, *p* > 0.05), while 3D-printed models did not improve knowledge acquisition.
Karsenty et al. [26]	RCT	347 medical students	CT	CHD including ASD, VSD, CoA and ToF.	Use of 3D printing improved objective knowledge when compared to the control group (*p* < 0.0001).
Smerling et al. [28]	Cross-sectional study	45 medical students	CT	Three-dimensional-printed heart models including pulmonic stenosis (PS), ASD, ToF, d-TGA, CoA and HLHS.	Three-dimensional-printed models significantly enhanced students’ knowledge for all of these cardiac pathologies (*p* < 0.001).
Su et al. [29]	RCT	63 medical students	CT	Three-dimensional-printed VSD models: perimembranous, subarterial and muscular VSD.	Three-dimensional-printed models significantly improved VSD learning when compared to the control group (*p* < 0.05).
Lau et al. [30]	Cross-sectional study	29 participants (radiologists, sonographers and radiographers)	CT	ASD, VSD, ToF and DORV.	Both 3D-printed models and VR were useful in education and pre-operative planning when compared to conventional visualisations.
Jones & Seckeler [31]	RCT	36 pediatric medical students	CT & MRI	Three-dimensional-printed models of vascular rings and pulmonary artery slings.	Three-dimensional-printed models significantly enhanced participants’ knowledge in learning anatomy and pathology (*p* < 0.01).
Loke et al. [19]	RCT	35 pediatric residents	CT, MRI and 3D echocardiography	Three-dimensional-printed models: A normal infant heart, an adult repaired ToF and an infant unrepaired ToF.	Higher satisfaction scores were achieved with 3D-printed models (*p* = 0.03).
Valverde et al. [33]	Non-RCT	127 participants	Echocardiography and CMR.	Value of 3D-printed models on medical education in criss-cross hearts.	Three-dimensional-printed models significantly increased knowledge in learning criss-cross heart anatomy (*p* < 0.001).
White et al. [34]	RCT	60 pediatric and emergency medicine residents	NA	Three-dimensional-printed models of four cases including VSD and ToF.	The 3D printing group scored significantly higher than the control group (*p* = 0.037) on ToF postlecture test, while the control group scored higher on the VSD postlecture test (*p* = 0.012).
Tan et al. [35]	RCT	132 nursing students	NA	Three-dimensional-printed model of a ASD case in teaching clinical nursing in congenital heart surgery.	Use of Three-dimensional-printed models significantly improved clinical nurses in learning congenital heart surgery.
Biglino et al. [54]	Cross-sectional study	100 cardiac nurses	NA	Nine 3D-printed models: a heathy heart, and other types of CHD.	Three-dimensional-printed models serve as useful tools in training adult and pediatric cardiac nurses for learning and understanding CHD.
Liang et al. [38]	Cross-sectional study	Expert group (n = 40 with 20 cardiac surgeons and 20 sonographers) and student group (40 postgraduate medical students)	CT	Eight types of CHD: PDA, CoA, VSD ccTGA, DORV, WA, CAF and ToF.	3D-printed models significantly improved the CHD diagnosis in both the expert and student groups (*p* = 0.000–0.001). Three-dimensional printing was found to be more important in the diagnosis of more complex CHD than simple CHD (*p* = 0.000).
Lee C and Lee J [55]	Cross-sectional study	74 participants including 41 residents, 14 physicians, 10 nurses and 9 perfusionists.	CT	Eleven complex CHD cases included ToF, AVSD, DORV, single ventricle with DILV/BCPA.	Subjective improvements were found in all learning categories post-seminar scores when compared with pre-seminar scores: understanding anatomy (8.4 ± 1.1 vs. 4.8 ± 2.1), 3D structure (8.9 ± 1.0 vs. 4.6 ± 2.2), pathophysiology (8.5 ± 1.0 vs. 4.8 ± 2.2), and surgery (8.8 ± 0.9 vs. 4.9 ± 2.3), with all *p* < 0.001 respectively.

ASD—atrial septal defect; AVSD—atrioventricular septal defect; BCPA—bidirectional cavopulmonary anastomosis; CAF—coronary artery fistula; CHD—congenital heart disease; CoA—coarctation of aorta; CT—computed tomography; CMR—cardiac magnetic resonance; ccTGA—corrected transposition of the great arteries; DILV—double inlet left ventricle; d-TGA—d-transposition of the great arteries; DORV—double outlet right ventricle; HLHS—hypoplastic left heart syndrome; MRI—magnetic resonance imaging; NA—not available; PDA—patent ductus arteriosus; RCT—randomized controlled trial; ToF—Tetralogy of Fallot; VR—virtual reality; VSD—ventricular septal defect; WS—William syndrome.

**Table 3:** Clinical value of 3D-printed CHD models in the surgical planning of CHD surgery. Modified from Sun and Wee [37].

Author	Study Design	Sample Size and Participants	Original Data Source	Application in CHD	Key Findings
Valverde et al. [40]	Prospective multicenter study	Forty patients with complex CHD.	CT and MRI	Three-dimensional-printed models of 19 DORV and 21 other types of CHD.	The surgical decision was changed in 47.5% cases with aid of 3D-printed models.
Chen et al. [63]	Cross-sectional study	Five patients with (PA with VSD or MAPCA	Echocardiography and CT	Three-dimensional-printed models and VR/MR in surgical outcomes.	Three-dimensional-printed models assisted surgeons to pre-operatively analyse surgery plans, while VR facilitated understanding of intracardiac structures.
Gomez-Ciriza et al. [21]	Cross-sectional study	Forty-three participants	CT and MRI	One hundred thirty-eight low-cost 3D-printed models were developed for surgical planning and interventional simulations.	Use of 3D-printed models has a positive impact on CHD surgery with initial surgical plan modified in 47.5% of the cases after reviewing the models.
Guo et al. [64]	Cross-sectional study	Surgeon, patients and nonmedical professionals	CT	Seven HOCM models were printed for surgical management and pre-operative conversation.	Three-dimensional-printed models were useful for surgical planning and pre-operative communication.
Kiraly et al [23]	Cross-sectional study	Single center team learning experience of 3D-printed models in pediatric surgeries	CT	Fifteen models of pediatric patients with CHD and their impact on complex CHD surgeries.	Three-dimensional-printed models significantly contributed to improved surgical plans with intracardiac repair modified in 13 out of 15 cases.
Ryan et al. [65]	Cross-sectional study	Single center experience of 3D-printed models in CHD	CT and MRI	One hundred sixty-four models were printed for various purposes.	Three-dimensional-printed models contributed to a mean reduction in overall time when compared with standard of care, although the reductions did not reach significant differences.
Zhao et al. [66]	Cross-sectional study	Twenty-five patients with DORV	CT	Use of 3D-printed models in pre-operative repair of complex DORV.	Three-dimensional-printed models significantly reduced operating time and improved postoperative outcomes (*p* < 0.05).
Ghosh et al. [67]	Cross-sectional study	Single center three-year experience with 112 3D-printed CHD models for pre-operative planning.	MRI and CT	Use of 3D-printed models in pre-procedural planning of CHD.	Demand for the use of 3D-printed models in clinical practice has tripled over a three-year period. Incorporation of 3D printing technology into pre-procedural care of pediatric CHD surgeries is feasible.

ASD—atrial septal defect; CHD—congenital heart disease; CT—computed tomography; DORV—double outlet right ventricle; HOCM—hypertrophic obstructive cardiomyopathy; MRI—magnetic resonance imaging; NA—not available; PA—pulmonary atresia; MAPCA—major aortopulmonary collateral arteries; Soc—standard of care; VR—virtual reality; VSD—ventricular septal defect.

**Table 4:** Clinical applications of 3D-printed models for HOST in congenital heart surgery procedures. Modified from Sun and Wee [37].

Author	Study Design	Sample Size and Participants	Original Data Source	Application in CHD	Image Processing Software	3D Printer	3D Printing Material	Key Findings
Yoo et al. [68]	Cross-sectional study	Fifty participants (surgeons and surgical trainees) participated in the survey.	CT and MRI	HOST using 3D-printed CHD models.	Mimics (Materalise, Belgium) Average cost per model: $60	Objet Connex 260 printer.	TangoPlus FullCure resin and VeroWhite	HOST serves as a valuable surgical simulation platform for practicing congenital heart surgery on 3D-printed models.
Hussein et al [85]	Cross-sectional study	Seven trainees completed 12 sessions through HOST program for congenital heart surgery.	NA	Twelve 3D-printed heart models were incorporated into year long currciulum.	NA	Polyjet (Stratasys J750, Eden Prairie, MN, USA).	Agilus30	Ninety-one percent of procedural times were improved by a mean of 25% (*p* < 0.001). Eighty-four percent of trainees’ mean time improved between the two attempts with an improvement of 23% (*p* = 0.002).
Scanlan et al [86]	Cross-sectional study	Four physicians and four pediatric cardiac surgery fellows assessed suitability of 3D-printed models for simulation of tricuspid valve annuloplasty and atrioventricular canal repair procedures.	Echocardiograpy	Three valve models (MV, TV and CAV) were printed with different materials for simulation of congenital heart valve procedures.	3D Slicer Directly printed model: $7.90 Molded: $45	Object 500 Connex (Stratasys, Eden Prairie, MN, USA)	Valve models printed in TangoPlus FLX 930, while valve mold printed in VeroGray RDG850 or VeroBlue GDG840	Surgeon assessment showed that the molded valve models were more realistic for cutting and suturing than directly printed models (*p* < 0.01). Complete atrioventricular canal repair was highly rated by surgeons using the molded valves compared with the directly printed valves (*p* < 0.01).
Hoashi et al. [88]	Cross-sectional study	Twenty models of CHD were created for surgical simulation with all operations performed by a young consultant surgeon.	NA	Understanding the relationship between intraventricular communications and great vessels and utility of 3D models for simulations of intracardiac procedures.	NA $2000–3000 per model	Stereolithography (SOUP2, 600GS, Japan)	Super flexible polyurethan resins	The median cardiopulmonary bypass time and cross-clamp time was 345 (110–570) min and 114 (35–293) min, respectively. No mortality was observed during the median follow-up of 1.3 (0.1–2.5) years.
Brunner et al. [87]	Cross-sectional study	Nineteen medical students and doctors participated in the hands-on training program.	CT	Hands-on training on simulation of interventional cardiology procedures on common CHD models.	Mimics (Materalise, Belgium)	Agilista 3200W Polyjet 3D printer	Silicone rubber	Practicing on 3D-printed models significantly reduced the mean fluoroscopy time and increased confidence in interventions on real patients.
Hon et al. [89]	Cross-sectional study	Fifteen preclinical medical students participated in the HOST course.	NA	Medical students rehearsed their knot-typing and simple suturing skills on 3D printed models.	NA	NA	Agilus30 (Stratasys, Eden Prairie, MN, USA)	All students were highly satisfied with 3D-printed models helping their understanding of CHD (4.80 ± 0.41) and learning complex anatomy (4.87 ± 0.35), with training sessions improving their assisting skills (4.93 ± 0.26).
Olivieri et al. [90]	Cross-sectional study	Seventy participants enrolled in the study including 22 physicians, 38 critical care nurses and 10 ancillary providers.	CT or MRI	Ten CHD cases were selected for cardiac surgery simulation.	Mimics (Materalise, Belgium)	Objet500 Connext (Stratays, Eden, Prairie, MN, USA)	Rigid plastic materials.	Three-dimensional-printed models were scored more useful (8.4 out of 10) than standard hands-off with 90% of participants scoring 8 out of 10 or higher.

CHD—congenital heart disease; CT—computed tomography; CAV—common atrioventricular valve; DORV—double outlet right ventricle; HLHS—hypoplastic left heart syndrome; MRI—magnetic resonance imaging; MV—mitral valve; NA—not available; TV—tricuspid valve.

## Data Availability

Not applicable.
